# CRISPR/Cas9 as a Mutagenic Factor

**DOI:** 10.3390/ijms25020823

**Published:** 2024-01-09

**Authors:** Andrey R. Shumega, Youri I. Pavlov, Angelina V. Chirinskaite, Aleksandr A. Rubel, Sergey G. Inge-Vechtomov, Elena I. Stepchenkova

**Affiliations:** 1 Department of Genetics and Biotechnology, St. Petersburg State University, 199034 St. Petersburg, Russia; shumega84@mail.ru (A.R.S.); ingevechtomov@gmail.com (S.G.I.-V.); 2 Eppley Institute for Research in Cancer and Allied Diseases, Fred and Pamela Buffett Cancer Center, University of Nebraska Medical Center, Omaha, NE 68198, USA; ypavlov@unmc.edu; 3 Departments of Biochemistry and Molecular Biology, Pathology and Microbiology, Genetics, Cell Biology and Anatomy, University of Nebraska Medical Center, Omaha, NE 68198, USA; 4 Center of Transgenesis and Genome Editing, St. Petersburg State University, Universitetskaja Emb., 7/9, 199034 St. Petersburg, Russia; chirinskaitea@yandex.ru; 5 Laboratory of Amyloid Biology, St. Petersburg State University, 199034 St. Petersburg, Russia; a.rubel@spbu.ru; 6 Vavilov Institute of General Genetics, St. Petersburg Branch, Russian Academy of Sciences, 199034 St. Petersburg, Russia

**Keywords:** genome editing, CRISPR/Cas9, mutations, off-target activity

## Abstract

The discovery of the CRISPR/Cas9 microbial adaptive immune system has revolutionized the field of genetics, by greatly enhancing the capacity for genome editing. CRISPR/Cas9-based editing starts with DNA breaks (or other lesions) predominantly at target sites and, unfortunately, at off-target genome sites. DNA repair systems differing in accuracy participate in establishing desired genetic changes but also introduce unwanted mutations, that may lead to hereditary, oncological, and other diseases. New approaches to alleviate the risks associated with genome editing include attenuating the off-target activity of editing complex through the use of modified forms of Cas9 nuclease and single guide RNA (sgRNA), improving delivery methods for sgRNA/Cas9 complex, and directing DNA lesions caused by the sgRNA/Cas9 to non-mutagenic repair pathways. Here, we have described CRISPR/Cas9 as a new powerful mutagenic factor, discussed its mutagenic properties, and reviewed factors influencing the mutagenic activity of CRISPR/Cas9.

## 1. Introduction

Recent advances in genome-editing technologies have revolutionized the field of fundamental and applied genetics and have opened up new avenues for treating previously considered incurable diseases. A common step of most genome editing approaches is the enzymatic cleavage of the genomic DNA, resulting in site-specific double-strand break (DSB). Editing occurs when the repair of the DSB results in a change in the DNA sequence at the target locus. Currently, there are four main classes of DNA nucleases that are used for targeting specific nucleotide sequence: Zinc Finger Nucleases (ZFNs), Transcriptional Activator-Like Effector Nucleases (TALEN), meganucleases, and Clustered Regularly Interspaced Short Palindromic Repeats (CRISPR)-associated protein 9 (Cas9) [1].

ZFNs are composed of a Fok1 restriction endonuclease domain and a series of custom-designed zinc finger domains that guide the nuclease to a specific genomic site [2]. Typically, ZFNs contain three or more zinc fingers, each recognizing a specific triplet of nucleotides. ZFNs work as dimers and can bind to target sequences of at least 18 bp long [3]. Similarly, TALENs are also homodimers; each monomer has two modules: Fok1 endonuclease domain and TALE DNA-binding domain, which is a bacterial effector and may be custom-designed for recognition of specific 12–20 bp target sequences [3]. The TALE DNA-binding domain is a tandem array of several 33–35 amino acid stretches [4]. Each repetitive motif recognizes only one specific nucleotide in DNA [4,5]. In practice, it is necessary to combine several DNA-binding repeats to obtain a construction that recognizes specific DNA sequences [3]. Both TALENs and ZFNs require the design and assembly of custom proteins, which makes them more complex and time-consuming to use than newer genome editing platforms based on CRISPR/Cas9 [3,6]. Homing endonucleases or meganucleases are natural enzymes. Meganucleases are considered the most specific endonucleases, as they are characterized by a large recognition site from 12 to 40 bp [7]. Meganucleases have been used for genome editing, but their use is limited by the difficulty in identifying and engineering new enzymes with desired specificities and a small repertoire of available meganucleases [3,8].

**Figure 1:**
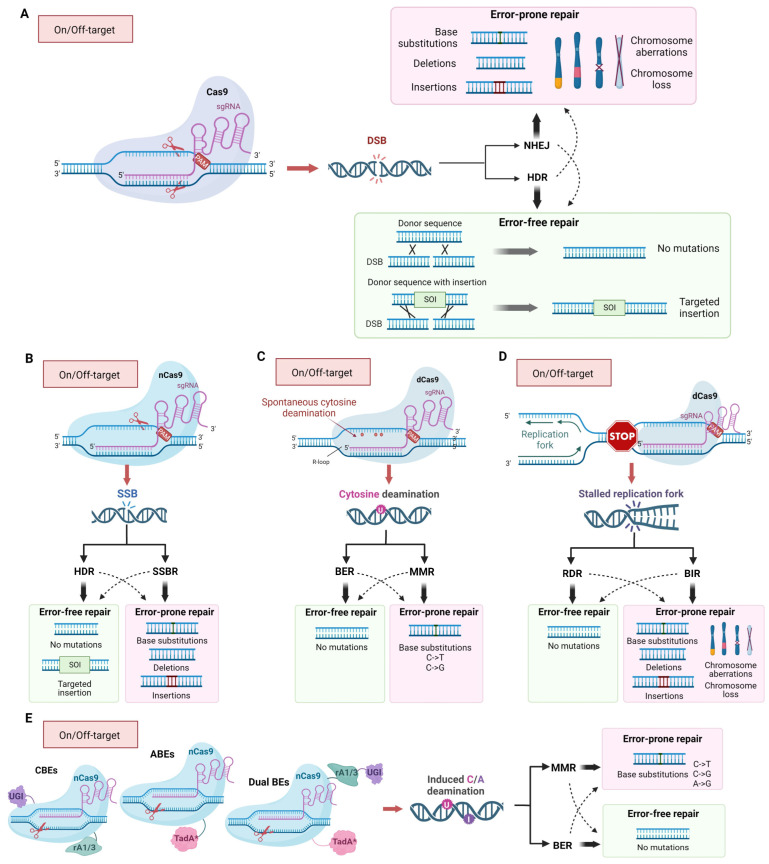
Mechanisms of mutagenic activity of CRISPR/Cas9 editing tools and its end-points both in on-target and off-target sites. (**A**) The inaccurate repair of double-strand breaks (DSBs) induced by the Cas9, mainly through non-homologous end joining (NHEJ), results in short indels and base substitutions, chromosome aberrations, and aneuploidy. Although HDR is more accurate than NHEJ, it may result in gene and chromosome mutations at a lower frequency. HDR and NHEJ can lead to accurate restoration of the DNA structure or, in the presence of a donor homologous sequence of interest (SOI), the insertion of homologous sequences can occur by homology-directed repair (HDR). (**B**) Modified nCas9 (nickase) cleaves only one of two DNA strands; the resulting single-strand brake (SSB) is the subject of HDR or SSB repair (SSBR), leading to targeted insertions, base substitutions, and deletions or insertions. (**C**) Spontaneous cytosine deamination in single-stranded DNA formed in the R-loop at the site of sgRNA/Cas9 binding. (**D**) Stalling of replication forks at sites where sgRNA/Cas9 interacts with DNA. (**E**) Chimeric complexes of dCas9 (catalytically inactive Cas9) and a DNA modifying enzymes (CBEs—Cytosine Base Editors; ABEs—Adenine Base Editors; Dual BEs—Dual Base Editors) deaminate cytosine or/and adenine and induce base substitution during replication or Base Excision Repair (BER) or Mismatch Repair (MMR). The bold and dashed arrows indicate major or minor repair pathways.

CRISPR/Cas9 is the most widely used genome-editing technology due to its simplicity, efficiency, and versatility. It is exploited in both fundamental research and practical applications. CRISPR/Cas9-based genome editing has been used to study gene function; in the development of gene therapies by correcting or replacing defective genes in various of cell types (including human embryonic and stem cells) [9,10]; in the discovery of new drug targets [11]; in pathogen diagnostics [12]; in genetic modification of plants to enhance crop yields, disease resistance, and reduce pesticide use [13]; and in other areas. The key element of the genome editing technology based on the CRISPR/Cas9 system is RNA-guided nuclease Cas9. It is capable of recognizing 20-nucleotide target sites and introducing double-strand breaks (DSBs) in genomic DNA within this sequence [14,15,16,17,18]. These breaks are then repaired mostly through either non-homologous end joining (NHEJ) or homology-directed repair (HDR) [19,20]. Genome editing occurs when DSB repair leads to the desired base substitution, deletion, or insertion (Figure 1A).

CRISPR/Cas9 systems pose a risk when used in medicine due to their ability to cause DNA-damage factors and potentially induce unwanted mutations both in on-target and off-target sites. The mutagenic activity of CRISPR/Cas9 is well established and relies on the accuracy of the DNA repair systems processing DSBs [19,20,21] since inaccurate repair of DSBs leads to mutational changes in DNA. Another factor influencing the genotoxicity of the CRISPR/Cas9 editing tools is off-target activity, which stems from the ability of the 20-nucleotide region of guide RNA to bind to sequences that may occur multiple times in the genome or sequences that differ from the target locus by a few nucleotides [21]. As a result, DNA breaks and mutations may occur in these off-target regions. Altered forms of Cas9 employed for genome editing can also cause damage to genetic material (Figure 1B). In addition, Cas9 binding per se can cause mutations through the spontaneous cytosine deamination in single-stranded DNA formed in the R-loop at the site of CRISPR/Cas9 binding [22] (Figure 1C) or through the stalling of replicative DNA polymerase, thereby causing large structural changes in the genome [23] (Figure 1D). Furthermore, unintended integration of exogenous sequences such as genomic DNA fragments [24], plasmids [25], and LINE-1 retrotransposons can also occur at the break sites [18]. CRISPR/Cas9-induced DSBs may lead to whole chromosome loss [10] (Figure 1A).

Since assessing the risks of using Cas9 for genome editing requires a thorough understanding of the mechanisms of its mutagenic action, we consider it is essential to analyze available data and take a look at Cas9 as a mutagenic factor and describe its key mutagenic properties, such as the type of DNA lesions caused by the editing complex, the repair systems involved in eliminating these damages, the type and frequency of arising mutations, and hot spots of mutagenesis.

## 2. Structure, Mechanism of Action and DNA Lesions Caused by CRISPR/Cas9 Editing Tools

Most CRISPR/Cas editing platforms originate from a natural system of adaptive immunity in bacteria and archaea responsible for cleaving foreign DNA that has entered the cell, e.g., plasmids or phages [26]. The formation of immunity, in this case, occurs in several steps. Upon entry, fragments of foreign DNA (known as spacers) are integrated into the CRISPR locus. When re-invasion occurs, transcription of the CRISPR locus begins, resulting in the production of a long molecule of pre-crRNA (pre-CRISPR-associated RNA), which is processed into short crRNAs (CRISPR-associated RNA). In the complex with Cas proteins, these short molecules bind to homologous segments of foreign DNA (known as protospacers), leading to the cleavage of the DNA by Cas proteins. Notably, the formation of a double-strand break in the protospacer DNA only occurs in the presence of a specific nucleotide sequence called the protospacer adjacent motif (PAM), which serves as a marker for foreign DNA [14]. The PAM sequences and their sizes vary among Cas nucleases, some examples for Cas9 are shown in Table 1.

**Figure 2:**
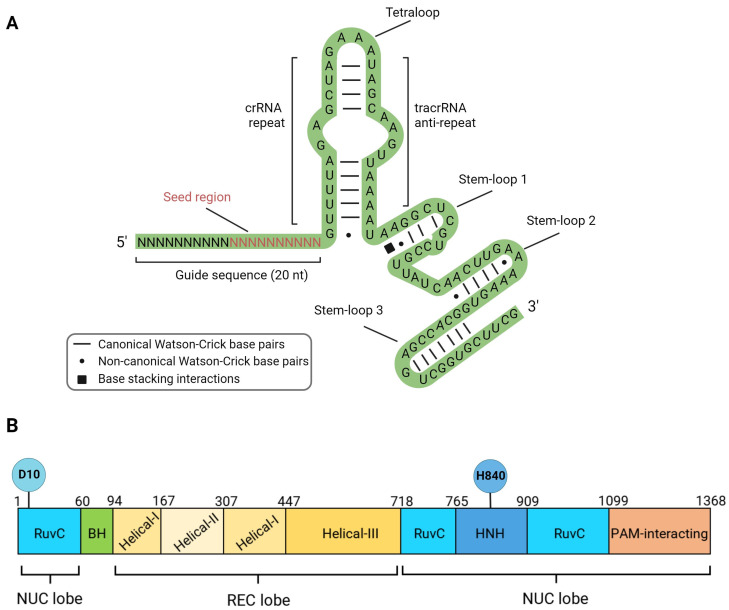
Components of active sgRNA/Cas9 editing complex. (**A**) Secondary structure of the sgRNA molecule: Guide sequence with 10 bp 3′ seed region; tracrRNA—trans-activating CRISPR RNA functional domain; crRNA—trans-activating CRISPR RNA functional domain. (**B**) Domain organization of the Cas9 protein. D10 and H840—amino acid residues essential for endonuclease catalytic activity. The nuclease (NUC) lobe is represented by endonuclease HNH and RuvC domains, the recognition (REC) lobe binds the nucleic acid and contains three recognition domains, Helical I–III.

All known natural CRISPR/Cas systems are divided into two classes and six types depending on the structure and mechanism of action [32]. The Class 1 systems are common in bacteria and are multi-protein types I, III, and IV complexes (Cascade, Cmr, Csm). The nuclease activity in Type I systems resides in the multi-protein Cascade complex associated with the Cas3 protein. Type III systems are characteristic of archaea, and nuclease activity is conferred by the multi-protein complexes Csm and Cmr. Type IV systems are relatively rare and poorly studied [33]. The Class 2 systems, on the other hand, have a single effector protein. This class includes types II, V, and VI. Type II systems are actively used in genetic engineering and are characterized by the presence of the endonuclease SpCas9 (*Streptococcus pyogenes*). SpCas9 (hereafter called Cas9) does not require additional protein cofactors for binding and cleaving target DNA. Inside the cell, two RNA molecules are required to activate Cas9: the CRISPR-associated RNA (crRNA), which contains fragments of foreign sequences, and the trans-activating crRNA (tracrRNA), which base pairs with crRNA and supports crRNA maturation. Cr-tracrRNA complex activates Cas9 through conformational changes. However, for simplicity in genetic engineering applications, both RNAs are combined into a single chimeric molecule called a single-guide RNA (sgRNA) [14]. The nucleoprotein complex used for genome editing consists of a sgRNA and the nuclease Cas9 (Figure 2). The sgRNA contains a sequence of 19–20 nucleotides (guide sequence) that is complementary to the target region of the genome, as well as functional domains tracrRNA and crRNA (Figure 2A). It is important to note that a ten-nucleotide segment from the 3’-end of the guide sequence (seed region) plays a crucial role in the recognition and cleavage of the target DNA [34].

The Cas9 protein consists of 10 domains, which are divided into two functional groups—the first group is responsible for DNA binding (helical recognition (REC) lobe), and the second group is responsible for introducing double-strand breaks (nuclease (NUC) lobe). The REC lobe includes the domains Helical I, Helical II, and Helical III, while the NUC lobe includes the domains RuvC-I, RuvC-II, RuvC-III, HNH, and PAM-interacting domain CTD [14] (Figure 2B).

The search for the target DNA and introduction of double-strand breaks occur in several stages: (1) Assembly of the sgRNA/Cas9 complex, resulting in a conformational change in the central part of the molecule, opening up DNA binding domains; (2) Stochastic interactions between the sgRNA/Cas9 complex and chromosomal DNA, which must contain the PAM sequence of the NGG type directly adjacent to the 3′-end of the sgRNA guide sequence; (3) Formation of hydrogen bonds between the CTD domain of Cas9 and PAM sequences distributed throughout the genome, and unwinding of the DNA duplex one nucleotide away from the PAM; (4) Complementary sgRNA promotes further unwinding of the target DNA duplex; (5) RuvC and HNH domains of the Cas9 protein introduce a double-strand break with blunt ends at a distance of three nucleotides from the PAM sequence [14]. Thus, the CRISPR/Cas9 editing system possesses a property characteristic of most mutagens—the ability to damage DNA.

The nucleoprotein complex sgRNA/Cas9 is widely used as a platform for developing various genome editing tools that cause DNA lesions of different types. Along with the wild-type protein capable of inducing a double-strand break, mutant variants of the nuclease are broadly used (Figure 1B,E). Two endonuclease domains of Cas9 cleave different DNA strands at a distance of 3 nucleotides from the 5′-end of the PAM sequence. The Cas9 HNH nuclease domain cleaves the strand complementary to the sgRNA, while the RuvC-like domain cleaves the non-complementary strand. Independent cleavage of each DNA strand leads to the DSB appearance [14]. Substitutions D10A (RuvC domain) or H840A (HNH domain) lead to the loss of the corresponding endonuclease activity (Figure 2) [14]. Cas9 with one inactivated endonuclease domain is called Cas9 nickase (nCas9); it introduces single-strand breaks (SSBs), which could result in mutations in the target DNA (Figure 1B). Inactivation of both endonuclease domains results in a catalytically dead enzyme (dCas9), that does not cleave DNA but serves as a delivery platform for targeting other enzymes fused with dCas9.

Both dCas9 and nCas9 may be fused with different functional protein domains to induce directed base substitutions without DSB introduction. Such complex enzymes are called base editors. Base editors can be divided into four main groups, Cytosine Base Editors (CBE), Adenine Base Editors (ABE), Dual-Base Editors (Dual BE), and Prime Editors (PE), depending on the mechanisms of editing. CBEs mostly consist of dCas9 or nCas9 nuclease conjugated with cytosine deaminases of the AID/APOBEC family and uracil glycosylase inhibitor (UGI) [35,36,37,38,39,40,41]. Upon binding to the target DNA sequence, deamination of cytosines leads to uracil formation in DNA. CBEs deaminate C to U at a distance of ~15 bp from the PAM motif [35]. Additionally, the “activity window” of the base editor, typically 5 bp in length, may contain several C that can be deaminated to U, leading to multiple C to T substitutions [40]. nCas9 fused with TadA adenine deaminase with altered substrate specificity (RNA-specific enzyme turned to DNA-specific via directed evolution) is called ABE. Due to TadA activity, adenine is turned to inosine in the ~11 to 16 position from PAM. DNA base modifications in template DNA strands are highly mutagenic because they lead to base substitutions during DNA synthesis (Figure 1E). nCas9-base fused with TadA and APOBEC deaminases simultaneously is called Dual BE. Such editors have detectable off-target activity, similarly to CBEs and ABEs, and editing efficiency is often lower than efficiency of CBE or ABE. Still, Dual BE is more versatile than CBE or ABE [42,43].

A completely new approach to DNA editing without inducing DSBs was presented in the article by Anzalone et al. [44]. The authors developed fusion enzymes called Prime Editors (PE), which consist of nCas9 and Moloney murine leukemia virus reverse transcriptase (RT). To guide PE to the desired genomic location, a special prime editing guide RNA (pegRNA) is used. The pegRNA contains two parts: a guiding fragment that directs nCas9 to the target site and an editing fragment that contains the desired DNA sequence changes at a distance ranging from 3 bp upstream to 29 bp downstream of a PAM. After nicking the PAM strand 3 bp upstream of PAM, a pegRNA–DNA duplex with non-target strand is formed, and then the pegRNA serves as a primer for RT, and DNA is synthesized. The newly synthesized DNA forms a 3′ flap that displaces the non-edited 5′-flap and then gets ligated in the genomic DNA. It is possible to obtain base substitutions, insertions, and deletions using PE. A few variations of PE were designed with off-target activity that was lower than that of spCas9 [44].

Each of Cas9 variants mentioned above is potentially mutagenic.

## 3. The Mechanism of Inaccurate Repair of Cas9-Induced DNA Lesions

There are two main mechanisms for repairing Cas9-induced DSBs—homology-dependent repair (HDR) and non-homologous end joining (NHEJ) (Figure 1A) [45,46,47]. However, minor pathways, such as microhomology-mediated end joining (MMEJ) [48] or break-induced replication (BIR) [49] may also be involved (Figure 1D). In general, the main factors determining the repair pathway are the DNA lesion’s nature and the cell cycle’s phase [50]. Repair of DSBs by NHEJ occurs during the G1 phase of the cell cycle, while HDR occurs during the G2/M phases. NHEJ often leads to genetic variability at Cas9 cleavage sites, as DNA exonucleases and low-fidelity DNA polymerases are involved in processing free DNA ends before ligation, increasing the likelihood of errors [51].

The initiation of NHEJ in mammalian cells occurs through binding to DNA ends in the double-strand break region by the Ku70/Ku80 heterodimer and DNA-dependent protein kinase (DNA-PK), which, together with the endonuclease Artemis, process the DNA ends (fragmentation, gap filling, removal of damaged nucleotides) for subsequent ligation. Then, the DNA ends are covalently joined by the DNA ligase IV/XRCC4 complex. Gap filling in DNA is carried out by polymerases λ and μ (PolX family), whose activation is mediated by the Ku/DNA complex through BRCT domains of the polymerases. Polymerases λ and μ are well suited for NHEJ as they are capable of synthesis without relying on a template [47].

In the case of homologous recombination, genetic information is restored using sister chromatids. In the presence of a transgenic DNA fragment with a locus homologous to a genomic region, recombination can occur between the transgene and the genomic DNA at the site of the double-strand break, resulting in the insertion of the transgenic sequence at the break site or replacement of the target sequence with the transgenic one. In the initial stage, end preparation is initiated at the double-strand break site by nucleotide excision in the 5’-3’ direction with the formation of single-stranded DNA (ssDNA). Then, one of the strands of the homologous sequence invades, forming a Holliday junction structure. In yeast *Saccharomyces cerevisiae*, it has been shown that the MRX complex (homologous to the MRN complex in mammals), which includes proteins Mre11, Rad50, Xrs2, and Sae2, is involved in processing DSBs ends [52]. In mammalian cells, DNA-dependent ATPase protein Rad51 plays an important role in DNA strand exchange, forming nucleoprotein filaments with DNA [53] and catalyzing strand exchange by forming D-loops. It is known that the protein p53 is responsible for activating the repair of double-strand breaks in mammalian cells. Phosphorylation of p53 occurs in response to DNA damage and initiates the transcription of double-strand break repair genes (*BRCA2*, *RAD51*, and *MRE11*). Phosphorylation of p53 is carried out by checkpoint kinases ATM and ATR, with ATM being a sensor of double-strand breaks [54].

The effectiveness of introducing genetic changes using sgRNA/Cas9 largely depends on the balance between homologous and non-homologous DNA repair [19,20,21], which changes during the cell cycle. NHEJ factors are expressed in cells throughout the cell cycle, while the activation of factors involved in homologous recombination occurs in the S/G2 phases of the cell cycle by increased expression and post-translational modifications of specific proteins [55]. This makes the S/G2 phases of the cell cycle the most favorable time for introducing desired genetic changes, as there is a higher likelihood of homology-directed repair occurring. The low frequency of homologous recombination is a significant challenge in genetic modification. For example, due to the high sensitivity of human pluripotent stem cells to genome damage, the frequency of modifications using CRISPR/Cas9 systems is less than 10% [56]. Promising approaches to enhance the efficiency of site-directed mutagenesis using CRISPR/Cas9 systems include altering the balance between homologous and non-homologous repair and activating the homologous mechanism in atypical cell cycle phases. One way to suppress the NHEJ mechanism is to inhibit key proteins. When short hairpin RNAs (shRNAs) targeting Ku70, Ku80, and DNA ligase IV are used, the frequency of homologous recombination increases up to 300% [57]. Additionally, when a small subunit of the protein Scr7, which blocks the DNA-binding domain of DNA ligase IV, and proteins involved in proteasomal degradation of DNA ligase IV (E1B55K and E4orf6) are added to shRNAs, the frequency of site-directed mutagenesis increases seven-fold compared to the baseline level [58]. Inhibition of another component of NHEJ, DNA-dependent protein kinase catalytic subunit (DNA-PKcs), increases the frequency of site-directed mutagenesis four-fold [59].

The Rad51 protein is a crucial element of homologous recombination, forming a nucleofilament on single-stranded DNA and participating in DNA duplex invasion. Treatment of cells with the compound RS-1 (4-Bromo-*N*-(4-bromophenyl)-3-[[(phenylmethyl)amino]sulfonyl]benzamide) stabilizes the binding of Rad51 to DNA and increases the frequency of site-directed mutagenesis by three to six times [60]. The treatment of pluripotent stem cells with nocodazole or ABT-751 stimulates their transition into the G2/M phases of the cell cycle, which enhances homologous recombination and consequently increases the frequency of site-directed mutagenesis by four times [61].

Modifications of the CRISPR/Cas9 gene editing platform that prevent DSB formation and induce DNA lesions of different types (single-strand breaks (SSB) and base alterations) have fewer side effects and higher accuracy, but they are still genotoxic. nCas9 and PEs could be a source of all types of mutations due to HDR and single-strand break repair (SSBR) (Figure 1B) [62]. The base editors CBE, ABE, and Dual BE, causing deamination of cytosine and adenine, provoke base substitutions (C to T and A to G) due to replicative DNA synthesis on a template, containing deaminated bases (Figure 1E). Additionally, deamination of cytosines may occur spontaneously in R-loops, formed by sgRNA and genomic DNA during editing. Deaminated bases are substrates for BER and MMR (Figure 1C) [63], which are potentially mutagenic multistep processes. In the first step of BER, uracil or hypoxanthine is excised by specific DNA glycosylases generating apurinic/apyrimidinic (AP) sites. AP sites are then converted to SSBs with an undamaged 3′-OH terminus and a base-free 5′-deoxyribose 5-phosphate residue at the 5′ end. Such lesions may stimulate DSBs formation [64,65] and further processed either through short-patch or long-patch pathways mediated by different DNA polymerases. In the short-patch pathway, the main role in filling gaps belongs to Pol β, but Pol λ also contributes to BER. Long-patch BER is mediated by Pol δ, or ε, that have high fidelity and fill longer gaps [66]. During MMR, single stand gaps are formed after the excision of unpaired or damaged nucleotides. Then, they are filled by Pol δ, or ε. Intermediates of SSBR, BER, and MMR are mutagenic because of limited fidelity of DNA polymerases, participating in gap filling during repair. Moreover, such BER intermediates, as AP sites are bypassed by TransLesion Synthesis (TLS) DNA polymerases, during replication when replication forks are stalled at the lesion. TLS is an error-prone process that leads to an increase in the rates of base substitutions, short insertions and deletions, complex mutations, and long deletions between repeating sequences [67,68,69]. By using different forms of Cas9 that cause specific types of DNA lesions, it is possible to shift the repair process toward a specific repair pathway and increase the likelihood of obtaining certain types of mutations. For example, it is well known that short insertions and deletions are the most common result of repair of Cas9-induced double-strand breaks. For the induction of longer deletions, Cas9 fused with human APOBEC3A, uracil DNA glucosidase, and apurinic or apyrimidinic site lyase may be used [70]. All the scenarios may take place at on- and off-target sites.

## 4. On-Target and Off-Target Activity: Hot Points of the sgRNA/Cas9-Dependent Mutagenesis

One of the important features of the sgRNA/Cas9 mutagenic activity is its high sequence specificity compared to other mutagenic factors. The high site-specific activity is determined by the presence of a PAM sequence and the subsequent 20 nucleotides complementary to the artificial sgRNA. The complex efficiency depends on the nucleotide at position N for the SpCas9 PAM (NGG), as it affects the change in the complex’s binding free energy with DNA and the stability of the sgRNA/Cas9 complex on genomic DNA. Therefore, a particular nucleotide at position N can have different effects on the activity of the editor [71]. However, it should be noted that the specificity of sgRNA/Cas9 is not absolute. The non-specific activity of sgRNA/Cas9 in off-target regions of the genome is one of the urgent problems, as it can lead to the emergence of pathogenic mutations, for example, in oncogenes or tumor suppressor genes. It is unclear why some off-target sites are cleaved by the Cas9 protein while others are not. The efficiency of sgRNA/Cas9 action can be affected by the chromatin structure of the target locus and by the presence of epigenetic modifications, such as DNA methylation or histone modifications [72,73,74]. In addition, the cell cycle stage and the level of DNA damage response can also affect the efficiency and specificity of sgRNA/Cas9 cleavage [75]. 

The level of expression and duration of action of editors in cells strongly influence the activity and accuracy of gene correction. Therefore, the delivery methods of sgRNA/Cas9 to target cells greatly influence its off-target effect [76,77,78,79,80,81]. Cas9 can be delivered into cells in the forms of DNA (plasmid transfection, viral transduction) or mRNA (microinjections, electroporation, or liposome-based vectors [82,83,84,85]), or it can be delivered into cells as sgRNA/protein complex (electroporation). In the latter case, the off-target activity is lower than in other delivery methods. For example, plasmid DNA poses an additional risk of insertional mutagenesis [86]. The expression of AAV-delivered genes (adeno-associated virus) persists for years in transfected cells [87,88]. Thus, AAV-mediated gene editing will likely yield unwanted on- and off-target mutations over time [89,90]. In contrast, Cas9 mRNA and sgRNA delivered by lipid nanoparticles (LNP) can be rapidly degraded in vivo, making LNP the most popular vector for in vivo gene editing [91].

Thus, for the sgRNA/Cas9 editing complex, hotspots of mutagenesis are sequences that are fully or partially complementary to the sgRNA. It is important to note that different sgRNAs, which are fully complementary to different sites in the genomic DNA differ in their ability to bind to the target sequence and, as a result, in the frequency of induced mutations. Since the delivery method and duration of exposure of the editing complex affect the frequency of mutagenesis, it can be concluded that this mutagenic factor, like other mutagens, exhibits a dose-dependent relationship.

The choice of guide sequence and the sgRNA/Cas9 complex’s design can influence its activity and specificity. The use of modified single guide RNAs or optimizing the PAM sequence can increase the specificity of sgRNA/Cas9 editing, while using modified Cas9 enzymes with reduced off-target activity can further improve its safety and accuracy. Various modifications of the Cas9 protein are often used to increase the activity and accuracy of editing. By using a nCas9 with two correctly matched sgRNAs, it is possible to obtain two close single-strand breaks that together produce a double-strand break [92,93]. The increase in specificity in the case of using paired sgRNA/Cas9-D10A complexes is about two orders of magnitude in the HEK-293FT cell line [93]. Increased specificity is also observed for protein variants eSpCas(1.0) (K810A K1003A R1060A) and eSpCas(1.1) (K848A K1003A R1060A). They made no off-target changes at 22 of the 24 predicted most likely off-target sites and were sensitive to one sgRNA mismatch outside the seed sequence [94]. In the highly specific nuclease SpCas9-HF1 with substitutions N497A R661A Q695A Q926A, the frequency of off-target mutations induced by SpCas9-HF1 was not statistically different from the spontaneous level of mutagenesis [95]. HypaCas9 variant (N692A M694A Q695A H698A) with mutations in the REC3 domain, is more specific compared to wt Cas9 and even to eSpCas(1.1) and SpCas9-HF1 and preserves similar efficiency [96]. Another variant, SuperFi-Cas9 (Y1010D Y1013D Y1016D V1018D R1019D Q1027D K1031D) showed a 500-fold increase in specificity [97], but its effect in cells has not yet been studied. Increased accuracy is also observed in enzymes evoCas9 (M495V Y515N K526E R661Q) [98], Sniper-Cas9 (F539S M763I K890N) [99] and HiFi Cas9 (R691A) [100]. Variants with multiple amino acid substitutions xCas9-3.6 E108G S217A A262T S409I E480K E543D M694I E1219V and xCas9-3.7 A262T R324L S409I E480K E543D M694 I E1219V, recognizing such PAM motifs as NG, NNG, GAA, GAT, and CAA, additionally exhibited in 10–100-fold higher target specificity in HEK-293T and U2OS cell lines [101].

Another factor influencing the non-specific activity of sgRNA/Cas9 is the number of nucleotide mismatches between the sgRNA guide sequence and the target chromosomal DNA, as well as the positions of the mismatches relative to the PAM sequence. It is assumed that the number of non-complementary bases between the guide sequence and a potential off-target sequence significantly affects off-target activity. The sgRNA/Cas9 complex can introduce double-strand breaks into sequences with incomplete homology to the guide sequence, containing up to four mismatched nucleotides [102] or even up to six contiguous mismatches [14]. Mismatches within a short sequence (8 to 12 nucleotides proximal to PAM) rarely influence off-target activity of the editor, while more distal mismatches relative to PAM lead to an increase in the frequency of off-target activity [15]. In other words, off-target activity correlates with the stability of the sgRNA/Cas9 complex on genomic DNA. An excess of potential energy in the interaction between the Cas9 protein and the PAM sequence of the target site can stabilize the Cas9-sgRNA-DNA complex when binding to an off-target site containing non-complementary bases. Firstly, it has been shown that the nuclease activity of Cas9 is activated after DNA strand unwinding at the target site [103]. In addition, the presence of non-complementary bases between the sgRNA guide sequence and the target DNA site within 1–12 nucleotides proximal to PAM suppresses the nuclease activity of Cas9. Secondly, certain nucleotides at positions 2, 3, 6, from the 5′ end of the sgRNA also negatively affect the activity of the complex. The 20th nucleotide from the 5′ end of the sgRNA preceding the PAM sequence is involved in initiating the unwinding of the DNA strand and stabilizing the Cas9-sgRNA-DNA complex in the PAM region. sgRNA molecules containing adenine at this position show a significant decrease in activity. Some nucleotides at positions 2 (Thymine), 3 (Guanine), and 6 (Adenine) also reduce the activity of the complex, despite the assumption that the 5ʹ end of the sgRNA (distal to PAM) is not essential for recognizing the genomic target site. Presumably, the decrease in complex binding efficiency to DNA is associated with weakened interaction between nucleotides 2, 3, and 6 and the Rec1 domain of the Cas9 protein [56]. It is also known that the efficiency of double-strand breaks is positively influenced by the optimal GC composition of the target sequence (40–60%), the ability to form secondary structures, and chromatin activity near the break (promoter regions) [56].

**Figure 3:**
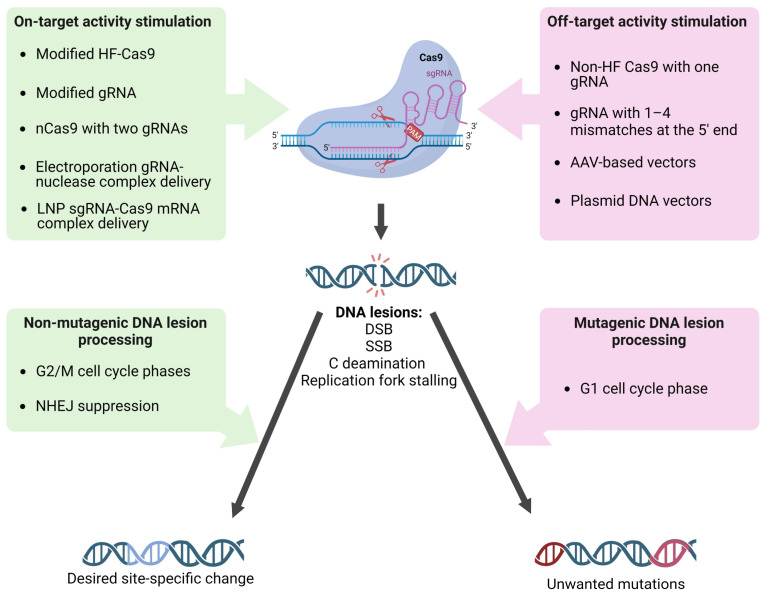
Factors influencing sgRNA/Cas9 targeting and DNA damage processing fidelity. The most accurate editing occurs when the factors listed on the left operate sequentially. Combinations of factors (on the right) that reduce the specificity of sgRNA/Cas9 binding to DNA and factors that direct lesions to mutagenic processing lead to the accumulation of unwanted mutations both in off- and target sites.

It has been shown that altering the secondary structure of the sgRNA molecule by adding two additional guanine nucleotides at the 5ʹ end leads to a decrease in non-specific activity without reducing target activity [104]. The addition of several cytosine nucleotides to the 5’ end of sgRNA significantly modifies the activity of the nucleoprotein complex. Polycytosine tails, depending on their length, reduce the cytotoxicity of the editing complex due to the low activation of p53, leading to the stimulation of homologous repair or a reduction in the on-target activity of the nuclease, improving the specificity and accuracy of monoallelic editing [105]. Shortening the sgRNA by three nucleotides leads to a decrease in the potential energy of complex binding to DNA, which significantly reduces off-target activity [106]. Chemical modifications of CRISPR RNA (crRNA) (Figure 2A) are also promising. As with other nucleic acid-based technologies, efforts are focused on sugar and backbone modifications (2′-deoxy, 2′-F, 2′-OMe, and phosphorothioates). Some more significant modifications of crRNAs have been made using bicyclic (locked) ribose and phosphate backbone substitutions (phosphonoacetates and amides); however, the range of chemical modifications applied to crRNA remains limited to modifications that have been successful in RNA interference and antisense technologies. Encouraging results on editing efficiency and accuracy have been obtained [107]. All of the above observations are actively used to design gene editing systems aimed at increasing efficiency and reducing off-target activity, including new target and guide sequence selection algorithms, new transfection methods, and new variants of Cas9/sgRNA modifications (Figure 3).

## 5. Types and Frequency of Mutations Caused by sgRNA/Cas9

When using CRISPR/Cas9 editing tools, their mutagenic properties are specifically applied to induce desired changes in the genome (Figure 3). First, sgRNA/Cas9 introduces a targeted DSB or other DNA lesion (SSB, cytosine, or adenine deamination). Then, various inheritable mutations occur with some probability during subsequent mutagenic repair of the damaged DNA. Moreover, during the repair of some lesions, the native DNA structure may be restored. By modifying repair pathways, it is possible to increase the proportion of the desired genetic changes. When the primary goal of genome editing is to inactivate a gene, in most cases, it is sufficient to introduce any convenient deletion or insertion in the gene’s coding region. In this case, researchers usually limit themselves to introducing a break and rely on selecting any random mutants that arise during break repair by NHEJ. However, when the goal is to make specific changes, the requirements for the accuracy of the editing process are significantly increased. It is unacceptable if unwanted sequence alterations accompany the required precise change. When obtaining a strictly defined mutation, a donor sequence with the desired mutation is introduced into the cell along with sgRNA/Cas9 to stimulate its integration in the genome through HDR (Figure 1A). However, editing accuracy is not absolute, and unwanted mutations can often occur, both at on-target and off-target sites, even during HDR (Figure 1). Cas9-induced DSBs in DNA can lead to the loss of chromosomes or their segments, and in the case of errors in NHEJ or HDR, small insertions/deletions (indel mutations) or point mutations and homologous replacements of target regions can occur, respectively [14] (Figure 1A). Mutations of all those types have been found in cells and organisms edited by sgRNA/Cas9 tools (Table 2).

An essential step in studying mutagens is to determine their specificity, or in other words, to identify all types of mutations that the mutagen causes and their frequencies. When studying on-target mutagenic activity, sequencing the target site in sgRNA/Cas9-treated cells is the most obvious method to choose. Detecting mutations in other areas of the genome is more challenging. The straightforward approach for identifying off-target mutations would be using high-throughput whole-genome sequencing (WGS). WGS has both advantages and limitations. With the use of WGS, the entire genome can be analyzed, and mutations of different types may be identified. However, WGS remains a relatively expensive procedure and could be less cost-efficient for detecting rare events. The success of WGS depends highly on the depth of coverage and proper choice and use of bioinformatic tools for data analysis. Therefore, efforts to develop new methods that could increase the accuracy of determining the frequency of mutations of different types at the genomic level continue. There are numerous methods used for predicting (E-CRISP, CRISPick, CHOPCHOP, CRISPR-ERA, CRISPOR, GUIDES, GeneArt, and uCRISPR) and detecting CRISPR/Cas9-mediated mutagenic activity at off-target sites (T7E1—endonuclease T7E1 cleavage of non-complementary DNA bases; IDAA—Indel Detection by Amplicon Analysis; WGS—whole-genome sequencing; Deep amplicon sequencing—high-throughput sequencing of amplicons; ChIP-seq—analysis of DNA–protein interactions based on chromatin immunoprecipitation (ChIP) and high-throughput DNA sequencing; GUIDE-seq—genome-wide identification of double-strand breaks based on high-throughput sequencing; HTGTS—high-throughput genome-wide translocation sequencing; and others) [118]. However, there is no single approach or small set of methods that significantly outperforms others in efficiency and sensitivity and optimized for the detection of at least the main classes of mutational changes in genetic material at once. The main problem stems from the fact that one of the stages of almost all the methods is the enrichment of potential editing sites, which are selected based on the analysis of the genome sequence using bioinformatics approaches. The accuracy of these tools is limited by the amount of experimental data available for designing the algorithms, leading to false positives or missed targets. Another concern is the low sensitivity of available methods for detecting off-target mutations, which does not exceed 0.01–0.1% (1 mutant per 10^3^–10^4^ wt cells or organisms) [119,120]. From classical genotoxic tests, it is known that the frequency of spontaneous point mutations in a reporter gene in haploid genomes is about 10^−8^–10^−6^, and strong chemical or physical mutagens induce this frequency up to two orders of magnitude [50,67,121,122]. Therefore, it is likely that the sensitivity of current methods used for studying off-target mutations in CRISPR/Cas9 edited cells does not allow the detection of mutations whose frequency is comparable to the frequency of mutations induced by classical mutagens.

There are several studies where the frequency of editing-induced mutations in on-target and off-target sites was estimated. In yeast *Saccharomyces cerevisiae*, Cas9 often causes +1 base pair insertions and other short indels up to 25 bps, and a minor fraction is base substitutions [108]. On-target mutations were found in up to 100% of clones. The authors used three reporter genes (*LYS2*, *CAN1*, and *MATα*), several different sgRNAs and several pairs of inverted PAM sequences (iPAMs). In the study, the frequency of each type of mutations depends on sgRNA used, differences were found even between two sgRNAs targeting a pair of iPAMs [108]. The authors also suggested a mechanism by which +1 insertions are formed: after cutting the PAM-containing strand at a distance of 4 from the PAM and the second strand at distance 3 bp, a 5′-overhang is formed in 1 nucleotide, which is filled in before ligating the ends. DNA polymerase 4 is required for most +1 insertions as well as for longer insertions [108]. In this study, mutation identification was performed using the Deep amplicon sequencing method on the Illumina MiSeq platform. The method includes DNA isolation, PCR of the target locus, library preparation, deep sequencing, and data analysis. This method allows the identification of mutations with the frequencies ranging from 0.01 to 0.1%. The disadvantage of this method is its high cost; moreover, it is a labor-intensive approach that may miss potential off-target sites in the genome [118,123].

When targeted modification of 63 immunity genes in tomatoes was performed, mutations were found in 245 out of 360 transformed plants (68%) [110]. The frequencies of mutagenesis (number of plants with target indel mutation/total number of transformed plants) for the 63 loci ranged from 14% to 100%. The most common mutational changes caused by sgRNA/Cas9 are deletions or insertions. Deletions or insertions of 1 bp are the most common of all mutations. For them, A- and T-inserts account for 79.5%, and G-inserts 4.5%. Base substitutions also occur but with much lower frequency [110]. However, the data obtained is not statistically significant since a small number of individuals were analyzed (from 1 to 29) depending on the locus due to the high labor intensity of agrobacterial transformation of tomatoes. Mutation identification was performed using site-specific PCR followed by Sanger sequencing [110].

The frequency of off-target mutations in plants was shown to be much lower than in mammals [111]. GUIDE-seq of 49 rice individual plants treated with sgRNA/Cas9 showed that most of the identified mutations (102–148 single-nucleotide polymorphisms (SNPs) and 32–83 indel mutations per plant) were the result of somaclonal variation (mutations, that arise frequently in regenerants of dedifferentiated plant cells). Off-target activity was not detected in 47 out of 49 analyzed plants. Similar data were obtained in studies with cotton [124] and maize [125]. All three studies examined small samples of plants and used the labor-intensive method of identifying genetic changes, GUIDE-seq. GUIDE-seq is based on integrating double-stranded end-protected oligodeoxynucleotides (dsODN) tags into sites of Cas9-induced double-strand breaks. Extracted genomic DNA is fragmented enzymatically or via sonication, and the resulting fragments undergo end-repair, dA-tailing, and ligation of a universal adapter sequence, which is added to both ends of all fragments. Target enrichment is achieved by two rounds of PCR that amplify only fragments containing the dsODN. Then, the resulting library is subjected to next-generation sequencing [126]. The disadvantage of the method is the presence of false negative results [127].

Recently, models based on protoplasts from leaf tissues of *Nicotiana benthamiana*, *Arabidopsis thaliana*, and other species have been used to study the nature of on-target and off-target activity of sgRNA/Cas9 in plants [128]. The advantages of such models include the increased sample size and simplified cell transformation of protoplasts.

In the study by Zhang et al. (2017) [112], the frequency of mutations in target regions was evaluated depending on the variant of the Cas9 nuclease used. For seven genomic regions, the frequency of on-target mutagenesis ranged from 8% to 25%. The frequency of off-target mutagenesis for individual loci ranged from 1% to 8%. Mutation identification was performed using the Deep amplicon sequencing method on the Illumina NextSeq 500 platform.

The frequency of off-target Cas9 activity was also analyzed in mice [114]. To assess the occurrence of Cas9-induced off-target mutagenesis, whole-genome sequencing with subsequent comparison of genomes of 50 Cas9-edited founder mice to 28 untreated control mice was carried out. Out of 26 WGS-detected Cas9-induced off-target mutant variants found in 15 founders, only 10 variants were later validated by Sanger sequencing with a resulting off-target mutation rate 0.2 Cas9 off-target mutations per founder analyzed. In comparison, there were more than 1000 unique non-Cas9-induced variants in the genomes of every mouse analyzed, indicating that Cas9 off-target mutations are a minor fraction of genetic heterogeneity in mice [114].

In a study by Yang et al. (2014) [115], knockout experiments of the Tafazzin (*TAZ*) gene in human embryonic stem cells were conducted. The sgRNA used effectively directed Cas9 to the target site, resulting in mutation in 54% of the cells. Using whole-genome sequencing, the authors mapped all possible off-target mutations that occurred due to editing the target site of the genome [115]. Thirty-one potential off-target sites, which differed from the target site up to three nucleotides, were tested. It was shown that the frequency of mutations occurring at all potential off-target sites did not exceed 0.15%.

In an experiment with human hematopoietic stem cells [117], T7E1 and GUIDE-seq were used. The T7E1 assay allows the detection of point mutations. The potentially mutable loci and the same sites in the unedited genome are amplified, and both amplification products are mixed, denatured, and renatured. If heteroduplexes have mismatches, they are cleaved by T7E1 endonuclease. Products of the reaction are visualized after agarose gel electrophoresis. The disadvantage of the method is that it is not 100% effective for some types of mismatches or bulges/distortions in DNA. Likewise, mutations that change primer binding sites will not be detected by the T7EI mismatch assay [129]. Also, elaborate bioinformatic prediction is necessary. With the use of T7E1 was coupled with the GUIDE-seq, it was shown that the frequency of specific mutagenesis (number of cells with target indel mutation/total number of transformed cells) was 59.07% for the *HBB* locus, 21.21% for the *ELANE* locus, and 58.64% for the *PRF1* locus.

In a review [130], 180 literature sources describing over 6400 off-target sequences in plants were analyzed to assess the influence of five factors on off-target activity: number of nucleotide mismatches, position of mismatches relative to the PAM sequence, GC composition of the target sequence, different variants of Cas9 nucleases, and delivery methods of the sgRNA/Cas9 complex. However, approximately 94% of the sequences represented cases with three or more nucleotide mismatches, in which off-target effects are rare events. Additionally, the studies used were highly diverse, including different plant species, transformation methods, methods for detecting genetic changes, and small sample sizes. Therefore, the evidence base in this case cannot be reliable and does not allow for unambiguous conclusions about the influence of specific factors on off-target activity.

Thus, editing tools based on sgRNA/Cas9 represent a mutagenic factor, the mutagenic activity of which requires a profound characterization due to its importance for safe use in medicine. In general, the molecular mechanisms underlying sgRNA/Cas9-induced mutagenesis are similar to the mechanisms of mutagenesis during the repair of DSBs induced by other factors. However, specific features of the formation and the repair of Cas9-induced breaks can affect the mutational profile of a CRISPR-edited genome.

## 6. Conclusions

CRISPR/Cas9-based DNA editing offers an exquisite opportunity for site-specific genome modification. However, its mutagenic activity needs to be thoroughly studied to ensure its safe use in medicine. On 8 December 2023, the U.S. Food and Drug Administration approved the first CRISPR-based treatment for sickle cell disease. More analogous treatments are in preclinical/clinical trials [131]. In the coming years, we can expect a surge in the development of new therapeutic approaches based on CRISPR technology because of the simultaneous emergence and improvement in critical areas, e.g., algorithms for target and sgRNA selection, efficient methods for RNP delivery, and new sgRNA/Cas9 modifications.

Completing research aimed at reducing off-target activity is the improvement of methods for assessing the frequency of mutagenesis induced by different sgRNA/Cas9 tools so that their sensitivity will be comparable with classical mutagenesis. Since CRISPR/Cas9 systems differ from typical mutagenic agents (chemical compounds and irradiation), standard methods used in genetic toxicology cannot be applied. A promising direction of studies is to compare the mutation rates in edited cells and cells treated with classical mutagens. For that, it is necessary to develop new in vivo models that allow large-scale studies to investigate the frequencies of on-target and off-target mutations. Classical model organisms, such as yeast *Saccharomyces cerevisiae*, are a logical choice for this purpose, allowing the generalization of statistically significant samples quickly and the exploitation of elaborated genetic, genetic engineering, and molecular biology methods [69,108,132].

It is essential to assess the mutagenicity of new modifications of genome editing technology at all stages of its development, both during basic research and at the stages of preclinical studies and clinical trials. This effort will help determine the threshold of mutagenesis frequency when Cas9 can be considered a safe technology. Thus, a considerable amount of research is devoted to approaches aimed at reducing the off-target activity of editing complexes. However, whether the achieved level is sufficient for safety needs to be clarified. Additionally, investigating the combined effect of CRISPR/Cas9 and other mutagens, such as some genotoxic therapeutic drugs is important. Comparing the mutagenicity of Cas9 in normal and cancer cells or cells with defects of DNA repair could be insightful, especially considering proposals to use genome editing to treat cancer. A significant problem is the influence of genetic heterogeneity of the human population on the accuracy of the selection of targets and putative sites of off-target activity in different ethnical groups or even individuals. Undoubtedly, with the increase in the number of new therapeutic approaches based on genome editing, there will be a need to standardize the procedure for assessing mutagenicity and establish thresholds for acceptable levels of mutagenesis in different tissues and organs treated with a genome editor. It may stimulate the development of universal methods, similar to what exists in traditional genetic toxicology, when during preclinical studies, standard tests are used to assess genetic safety, such as the Ames test, assessment of chromosomal aberrations in peripheral blood cells, and micronucleus test [133]. While achieving a universal method for all editing complexes may be challenging, it sets a goal for future research.

A comprehensive understanding of the factors influencing the mutagenic activity of CRISPR/Cas9, coupled with ongoing efforts to enhance the precision and safety of the technology, will contribute to its responsible and effective use in medicine and other fields.

## Figures and Tables

**Table 1:** PAM sequences in various nuclease families.

Nuclease	Species	PAM Sequences (5′ to 3′)
SpCas9	*Streptococcus pyogenes*	NGG [14]
SaCas9	*Staphylococcus aureus*	NNGRRT or NNGRR [27,28]
NmeCas9	*Neisseria meningitidis*	NNNNGATT [29]
CjCas9	*Campylobacter jejuni*	NNNNRYAC [30]
StCas9	*Streptococcus thermophilus*	NNAGAAW [31]

N—any base; R—purines; Y—pyrimidines; W—adenine or thymine.

**Table 2:** Different types of mutations induced by sgRNA/Cas9 in different organisms.

Organism	Location	Mutation Type	Frequency
*Saccharomyces cerevisiae* [108]	On-target	+1 bp insertions	0–74%
		+2 bp insertions	0–22%
		+3 bp	0–9%
	Off-target	No data	
*Caenorhabditis elegans* [109]	On-target	Chromosomal translocation	12.6%
	Off-target	No data	
Tomato [110]	On-target	A- and T-inserts	79.5%
	Off-target	18 predicted off-target sites	0%
Rice [111]	On-target	7 gene indels	15–100%
	Off-target	Indels, SNVs	0%
Rice protoplasts [112]	On-target	Indel mutations	25%
	Off-target	Indel mutations	1–8%
Mice [113]	On-target	Single-base substitution G>A in the *Gnao1* gene	25%
	Off-target	10 predicted off-target sites	0%
Mice [114]	On-target	Exon deletion	98%
	Off-target	Indels, single-nucleotide variants (SNVs)	0.2%
Human embryonic stem cells [115]	On-target	1–4 base pair deletions Tafazzin (*TAZ*) gene	54%
	Off-target	Indel mutations	0.15%
Human T-cells [116]	On-target	Loss of chromosomes 14 segment	20%
	Off-target	No data	
Human hematopoietic stem cell lines [117]	On-target	Indel mutation of *HBB* locus	59.07%
		Indel mutation of *ELANE* locus	21.21%
		Indel mutation of *PRF1* locus	58.64%
	Off-target	No data	
HEK293T, HeLa, and U2OS [18]	On-target	Insertions of LINE-1 (L1) retrotransposons	4–6%
	Off-target	No data	

## Data Availability

No new data were created or analyzed in this study. Data sharing is not applicable to this article.
